# Primary Peripheral Epstein-Barr Virus Infection Can Lead to CNS Infection and Neuroinflammation in a Rabbit Model: Implications for Multiple Sclerosis Pathogenesis

**DOI:** 10.3389/fimmu.2021.764937

**Published:** 2021-11-25

**Authors:** Asma Hassani, Narendran Reguraman, Safa Shehab, Gulfaraz Khan

**Affiliations:** ^1^ Department of Medical Microbiology & Immunology, College of Medicine and Health Sciences, United Arab Emirates University, Al Ain, United Arab Emirates; ^2^ Department of Anatomy, College of Medicine and Health Sciences, United Arab Emirates University, Al Ain, United Arab Emirates; ^3^ Zayed Center for Health Sciences, United Arab Emirates University, Al Ain, United Arab Emirates

**Keywords:** EBV - Epstein-Barr virus, peripheral infection, neuroinflammation, demyelination, multiple sclerosis, rabbit model, CNS infection

## Abstract

Epstein-Barr virus (EBV) is a common herpesvirus associated with malignant and non-malignant conditions. An accumulating body of evidence supports a role for EBV in the pathogenesis of multiple sclerosis (MS), a demyelinating disease of the CNS. However, little is known about the details of the link between EBV and MS. One obstacle which has hindered research in this area has been the lack of a suitable animal model recapitulating natural infection in humans. We have recently shown that healthy rabbits are susceptible to EBV infection, and viral persistence in these animals mimics latent infection in humans. We used the rabbit model to investigate if peripheral EBV infection can lead to infection of the CNS and its potential consequences. We injected EBV intravenously in one group of animals, and phosphate-buffered saline (PBS) in another, with and without immunosuppression. Histopathological changes and viral dynamics were examined in peripheral blood, spleen, brain, and spinal cord, using a range of molecular and histopathology techniques. Our investigations uncovered important findings that could not be previously addressed. We showed that primary peripheral EBV infection can lead to the virus traversing the CNS. Cell associated, but not free virus in the plasma, correlated with CNS infection. The infected cells within the brain were found to be B-lymphocytes. Most notably, animals injected with EBV, but not PBS, developed inflammatory cellular aggregates in the CNS. The incidence of these aggregates increased in the immunosuppressed animals. The cellular aggregates contained compact clusters of macrophages surrounded by reactive astrocytes and dispersed B and T lymphocytes, but not myelinated nerve fibers. Moreover, studying EBV infection over a span of 28 days, revealed that the peak point for viral load in the periphery and CNS coincides with increased occurrence of cellular aggregates in the brain. Finally, peripheral EBV infection triggered temporal changes in the expression of latent viral transcripts and cytokines in the brain. The present study provides the first direct *in vivo* evidence for the role of peripheral EBV infection in CNS pathology, and highlights a unique model to dissect viral mechanisms contributing to the development of MS.

## 1 Introduction

Epstein-Barr virus (EBV) is a B cell-tropic DNA virus belonging to the *Herpesviridae* family. The virus is often acquired early in childhood and then persists asymptomatically for life. EBV spreads from one host to another through virions intermittently shed in the saliva of infected hosts. The virus infects B lymphocytes *via* the interaction of the viral glycoprotein gp350/220 with the cell surface receptor, CD21 (1). Latently infected cells, express a range of viral genes referred to as latency programs 0-III. Cells in latency III express 6 nuclear antigens (EBNAs), 3 latent membrane proteins (LMPs), a set of viral encoded miRNAs, and 2 non-coding RNAs (EBERs) (2). EBERs are ubiquitously expressed in all forms of latency and are often used as targets for the detection of EBV in tissues. Collectively, latent viral proteins expressed during latency III appear to be fundamental for EBV transforming capacity (3). In the face of a competent immune response, EBV shuts down the expression of all viral genes, with the exception of EBERs (4). Despite the predominance of the latent cycle, EBV infected cells occasionally undergo acute lytic replication, which aids the dissemination of the virus. Moreover, the lytic cycle contributes to transient viremia, and increased peripheral viral load during the acute phase of infection (5).

Acquisition of EBV during late adolescence or early adulthood can lead to symptomatic infectious mononucleosis (IM) (6), which is an important risk factor for the development of multiple sclerosis (MS) (7). MS is a disease that results in the destruction of myelin sheaths in the brain and spinal cord, a process known as demyelination. The influx of inflammatory immune cells and reactive gliosis are other important hallmarks of MS (8). In addition to demyelination and inflammation, EBV infection in the brain has been reported in MS cases (9–12). However, it is unclear how peripheral EBV travels to the CNS and what its consequences are on the CNS.

The CNS is no longer considered an immune privileged site, as once thought. Rather, there is a bi-directional intricate communication between the periphery and the CNS. Inflammation in the periphery can interfere with the blood-brain barrier (BBB) integrity and induce changes in the brain (13, 14). Similarly, when murine γ-herpesvirus 68 (MHV-68), a virus that naturally infects rodents and is biologically similar to EBV, is introduced directly into the brain of BALB/c mice, the virus can spread from the site of inoculation (i.e. brain) to the peripheral organs, including the spleen. Moreover, on reactivation of latent virus, MHV-68 can be readily detected in both the CNS, and the spleen (15).

Our laboratory has previously shown that intravenous inoculation of rabbits with EBV results in the virus establishing latency that mimics asymptomatic infection in humans (16). Upon primary infection, rabbits elicited a strong humoral response, correlating with undetectable levels of the virus in peripheral blood. However, immunosuppression of latently infected animals using cyclosporin A (CsA), resulted in reactivation and marked increase in peripheral viral load. EBV reactivation was associated with the expression of the immediate early lytic marker, BZLF1, and a handful of latent viral genes. These animals also showed pronounced infiltration of infected cells into the liver and the spleen (16).

In this study, we aimed to understand the impact of peripheral EBV infection on the CNS in the rabbit model. We proposed that latent EBV infection in rabbits could promote pathological alterations in the CNS that may predispose the infected animals to features seen in MS, such as inflammation and demyelination.

## 2 Materials and Methods

### 2.1 Ethical Statement

All animal procedures in this study were reviewed and approved by the Institutional Review Board. Experiments were conducted on animals in adherence to the protocols approved by the Animal Research Ethics Committee of UAE University (Approval numbers: A-15-15; ERA-2018-5718).

### 2.2 Preparation of Virus Inoculum

B95-8, a B cell line of marmoset origin, was used to produce EBV for inoculation. The cells were grown in RPMI-1640 medium (GIBCO, USA) supplemented with 10% fetal bovine serum (FBS), 100 U/ml penicillin-streptomycin solution (GIBCO, USA), 50 µg/ml gentamycin (Hyclone, USA), and 1× glutamine (GIBCO, USA) at 37°C, and 5% CO_2_. Cells were then stressed by incubating at 30°C for 24hr to stimulate lytic cycle and virus shedding into the supernatant. The supernatant was centrifuged, and subsequently passed through 0.2-μm nylon filter (Thermo Fisher, USA). The filtered supernatant was used for intravenous (IV) injections following quantification of EBV copy number using qPCR.

### 2.3 Animals and Experimental Design

This study was divided into 2 parts:

a) investigating viral spread from the periphery to the CNS
b) investigating the dynamics of EBV infection over time

#### 2.3.1 Investigating Viral Spread From the Periphery to the CNS

Four- to 8-week old New Zealand White (NZW) rabbits were obtained from a local supplier, and housed in our animal facility in the College of Medicine and Health Sciences (UAE University). Following a 2-week acclimatization, a total of 24 rabbits were randomly allocated to four groups (**Supplementary Figure 1A**):

**Group 1 (EBV):** eight animals were injected with 1×10^7^ EBV copies, as determined by qPCR, *via* the marginal ear vein.
**Group 2 (PBS control):** four animals were IV injected with phosphate-buffered saline (PBS) (volume equivalent to that of EBV inoculum).
**Group 3 (EBV+CsA):** nine animals were injected with the same viral inoculum as for group 1 and treated with daily subcutaneous injections of cyclosporin A (CsA), (20mg/kg body weight, Sandimmune- Novartis).
**Group 4 (CsA control):** three animals were IV injected with PBS and immunosuppressed using daily CsA injections as in group 3.
Rabbits were monitored on a daily basis and sacrificed at day 14 post inoculation under Ketamine-Xylazine (40mg/kg and 5mg/kg, respectively) anesthesia. Whole blood was collected and separated into peripheral blood mononuclear cells (PBMCs) and plasma. Major organs including the spleen, brain and spinal cord were harvested.

#### 2.3.2 Investigating the Dynamics of EBV Infection Over Time

In this set of experiments, NZW rabbits were divided into EBV group and PBS control group. At day 0, 15 animals in EBV group received the virus, and five animals in the control group received PBS *via* IV injection as described above. Three randomly selected rabbits from the EBV group and one rabbit from the control group were sacrificed at each of the following five time points: 3, 7, 14, 21 and 28 days post inoculation (**Supplementary Figure 1B**). Whole blood, spleen, brain, and spinal cord were collected.

### 2.4 DNA Extraction and qPCR for EBV Genome

PBMCs and plasma were isolated from whole blood samples using density gradient centrifugation on Histopaque-1077 (Sigma, Poole, UK). Genomic DNA (gDNA) was extracted from PBMCs, plasma, and biopsied tissues from spleen, brain, and spinal cord using QIAamp DNA Mini and Blood Mini Kit (QIAGEN), according to manufacturer’s instructions.

Quantitative TaqMan PCR (Applied Biosystems) amplifying EBV BamHI fragment (17) was used to determine EBV copy number as previously described (18). The amplification reactions were run in duplicates on an Applied Biosystem 7500 real time thermocycler (Applied Biosystems). gDNA extracted from Namalwa cells was used to create a standard curve. Samples with undetermined Ct values were interpreted to have zero copy number for the purpose of statistical analysis.

### 2.5 RNA Extraction and RT-PCR

Total RNA was extracted from the brain using TRizol (Invitrogen, Germany). After determining the quantity and quality of the extracted RNA using NanoDrop 2000c (Thermo), 1µg of DNase-treated RNA was reverse-transcribed to cDNA using the Reverse Transcription System (Promega). SYBR Green Real-time PCR was performed, using Applied Biosystems QuantStudio™ 7 Flex System, to determine the relative mRNA expression of tumor necrosis factor α (TNFα), interleukin-1β (IL1β), IL2, and IL6 (19). The relative expression of latent EBV transcripts, EBER1, EBER2, EBNA1, and EBNA2 were also determined (20, 21). Samples were run in duplicates, and experiments were repeated twice. Relative expression was determined using comparative CT (ΔΔCt) method. Rabbit-specific GAPDH (housekeeping gene), and non-infected PBS samples (experimental controls) were used as reference.

### 2.6 Histology, EBER *In Situ* Hybridization (EBER-ISH), Immunohistochemistry, and Immunofluorescence

Formalin-fixed, paraffin-embedded (FFPE) tissues were cut into 5-μm sections and stained with hematoxylin and eosin (H&E) for basic histological examination. To identify viral proteins and cell populations contributing to inflammation in the CNS, a number of primary antibodies for viral and cellular markers were used (**Supplementary Table 1**).

For EBER-ISH, tissues were hybridized with a combination of 2 digoxin end-labelled probes complementary to EBER1 and EBER2, as previously described (18). Following blocking of the endogenous peroxidase activity, tissues were briefly digested with 0.1mg/ml proteinase K (Sigma). Sections were hybridized overnight with the probes, and two stringency washes were performed in 0.1×SSC buffer at 55°C. Mouse anti-digoxin antibody and Ultra-Sensitive ABC-Peroxidase Staining kit (Thermo Scientific, USA) were used for signal detection.

For chromogenic immunohistochemistry, heat-induced antigen retrieval was performed by incubating sections in boiling sodium citrate buffer (pH 6.2) for 10min. Endogenous peroxidase activity was quenched, followed by blocking in 5% BSA and 0.1% Triton-X 100 in 1×PBS for 1hr. Tissues were then incubated with primary antibodies at room temperature, overnight. Tissues were washed and incubated with appropriate secondary antibodies for 1hr. Diaminobenzidine (DAB) was used for signal detection and sections were counterstained with hematoxylin.

For immunofluorescence, sections were incubated with primary antibodies overnight. After washing, sections were incubated with fluorochrome-conjugated secondary antibodies for 1hr. Sections were washed, counterstained with DAPI and mounted using Fluoromount (Sigma). Fluorescence images were captured using fluorescence microscope (Zeiss).

For EBER-FISH and immunofluorescence, sections were hybridized overnight with EBER probes. After stringency wash, sections were incubated with goat anti-IgG for 1hr. Fluorochrome-conjugated anti-digoxin and anti-goat antibodies were used as secondary antibodies.

### 2.7 Protein Extraction and ELISA

Homogenates of brain cortex were prepared in T-PER tissue protein extraction reagent (Thermo) and proteinase-inhibitor cocktail (Roche), using BeadBlaster™24 Homogenizer (Benchmark). Purified proteins were stored at -80°C until analysis. DuoSet ELISA development system for rabbit IL2 and IL6 were used according to the manufacturer’s instructions (R&D Systems). All samples were assayed in duplicates, and experiments were repeated 3 times. A standard curve was included in each experiment and used to determine the quantity of cytokines in the test samples.

### 2.8 Statistical Analysis

Statistical analyses were performed using the GraphPad Prism Version 9.1.2 (GraphPad Software, San Diego, CA). Comparison between multiple groups was performed using one-way ANOVA or non-parametric multiple comparison, alpha= 0.05. Comparison between two groups was done using two-tailed unpaired t-test or nonparametric Mann-Whitney test. Data was displayed as mean± SEM. Spearman or Pearson correlation was used to correlate between 2 variables. *P* value ≤ 0.05 was considered statistically significant.

## 3 Results

### 3.1 Investigating Viral Spread From the Periphery to the CNS

#### 3.1.1 EBV Inoculated Rabbits Exhibit Viremia and High Viral DNA Load in the Periphery

The presence of EBV in MS brain has been reported in several studies (9–11, 18). How EBV in the periphery travels to the CNS is poorly understood. In order to determine whether peripheral EBV infection on its own can lead to CNS infection, we injected EBV intravenously into eight healthy NZW rabbits. In healthy animals, antiviral T cell responses act as a barrier that limits systemic viral dissemination. Therefore, in another nine rabbits, we injected EBV and immunosuppressed them using CsA. This was implemented to overcome anti-EBV T cell responses and increase the likelihood of EBV spreading to the CNS in these animals.

In the eight animals inoculated with EBV, no disease manifestations were observed during the 14-day study period, or at autopsy. In the EBV+CsA group however, 1/9 rabbits showed changed temperament, decreased activity, and major loss in body weight. To minimize animal suffering, this rabbit was euthanized at day 7, as opposed to the scheduled day 14. At autopsy, the spleen was found to be significantly enlarged with macroscopic white nodules in 4/9 rabbits in the EBV+CsA group, but not in any of the eight animals in the EBV group.

**Figure 1:**
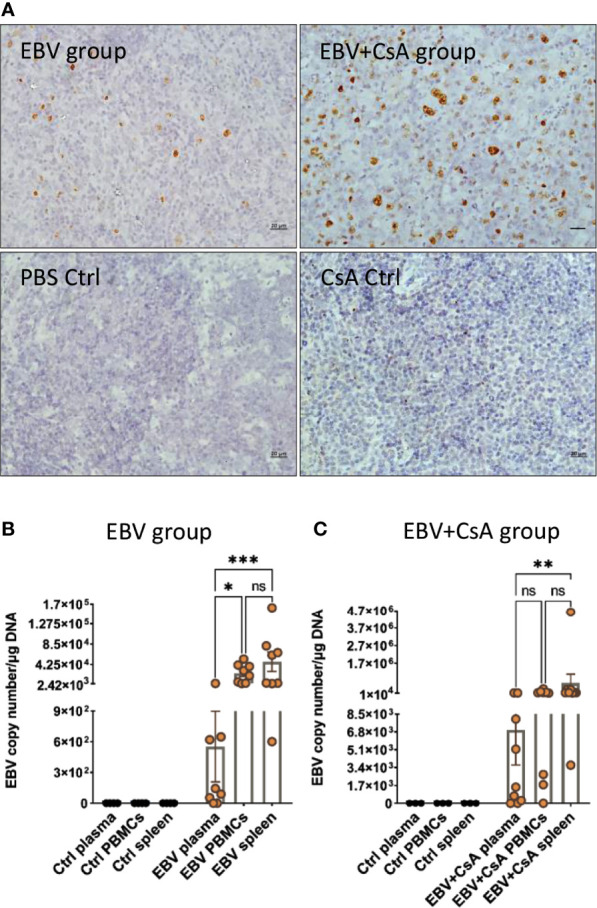
EBV inoculated rabbits exhibit viremia and high viral DNA load in the periphery. **(A)** Representative images of EBER-ISH on rabbit spleen from EBV and EBV+CsA group and their corresponding controls. Scale bar=20µm. **(B)** EBV copy number measured using qPCR in plasma, PBMCs, and spleen of rabbits in EBV group (*n*=8) and PBS controls (*n*=4), and **(C)** EBV+CsA group (*n*=9) and CsA controls (*n*=3). Samples with undetermined EBV levels were plotted at *y*=0. EBV load is presented with mean ± SEM, and comparisons were made using the nonparametric Friedman test and Kruskal-Wallis. ns: *p >* 0.05, *p ≤ 0.05, **p ≤ 0.01, ***p ≤ 0.001.

EBV infection in the spleen was confirmed by EBER *in situ* hybridization (EBER-ISH) in all animals inoculated with the virus. No EBER signal was seen in the spleen of any of the control rabbits that were injected with either, PBS or CsA (**Figure 1A**). This was further supported by the detection of the virus using qPCR. EBV genome was detected in the spleen of all the rabbits in both EBV and EBV+CsA groups (**Figures 1B, C**). Thus, peripheral EBV infection was established in 100% of the animals inoculated with EBV, regardless of the immune status.

Since viremia is an important event in predisposing to CNS infection (22), we quantified EBV in the PBMCs and plasma. All the animals in the EBV group (8/8) (**Figure 1B**) and 8/9 animals in EBV+CsA group (**Figure 1C**) had quantifiable, but variable viral load in the PBMCs. None of the PBS/CsA controls had detectable virus (**Figures 1B, C**). In the EBV+CsA group, the 1/9 animals which did not have detectable virus in the PBMCs, was the animal which was euthanized 1 week prematurely. This suggests that EBV detectability in peripheral blood may be suboptimal at 7dpi as opposed to 14dpi. As for plasma, 75% (6/8) of the animals in the EBV group (**Figure 1B**) and 78% (7/9) of EBV+CsA group (**Figure 1C**) were viremic (i.e. EBV DNA in plasma). We also found significant correlation between the levels of viremia and splenic viral load in the EBV group, but not in the EBV+CsA group (**Supplementary Table 2**). This implies the interfering effects of immunosuppression on viremia levels. Among the 3 peripheral compartments (plasma, PBMCs and spleen), plasma had the lowest level of EBV DNA (**Figures 1B, C**). This indicates that lytic shedding of the virus is relatively less frequent, and most of the virus is cell-associated.

#### 3.1.2 Inflammatory Cell Aggregates Develop in the CNS Following Peripheral EBV Infection

**Figure 2:**
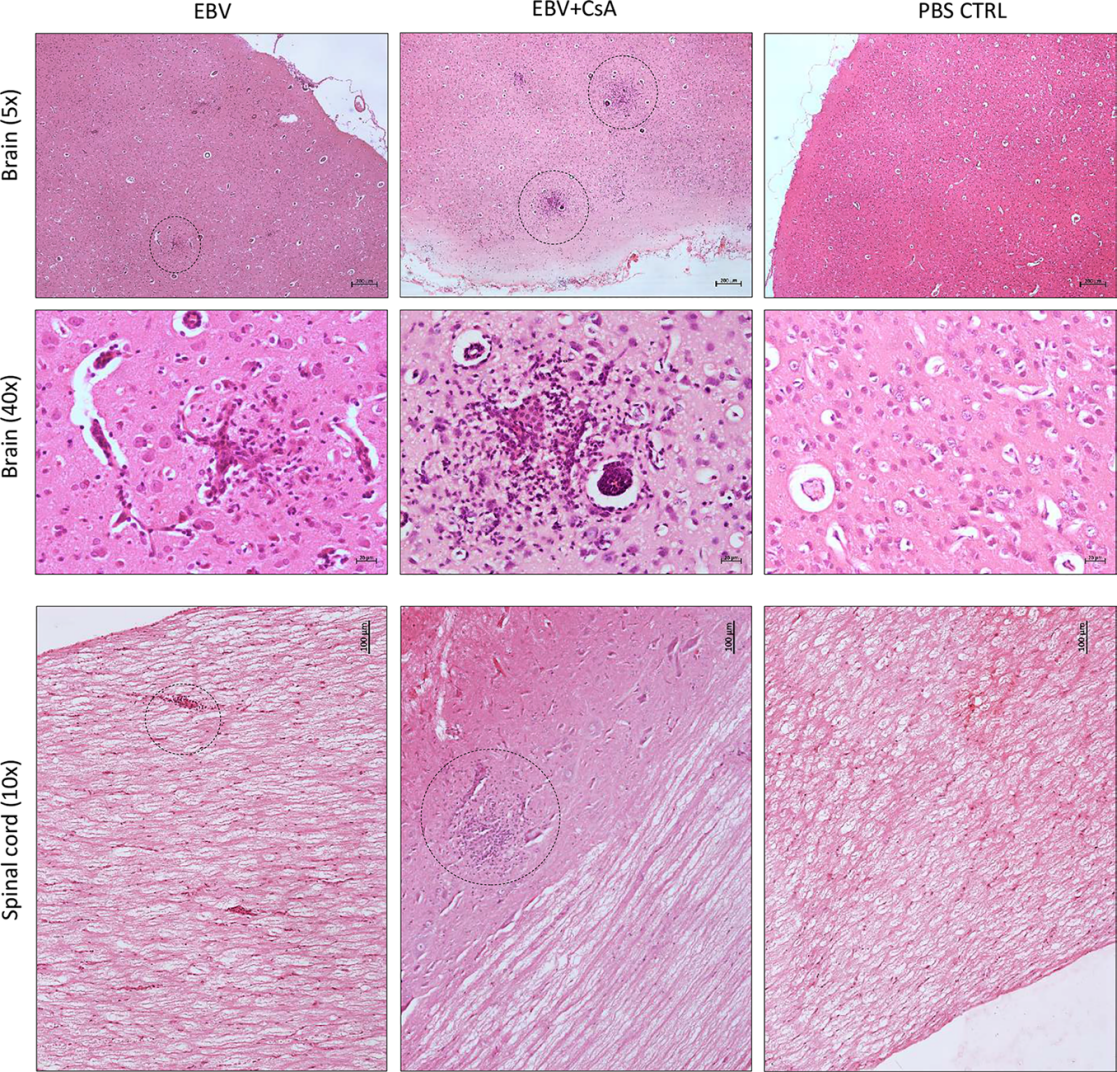
Cell aggregation in the rabbit CNS. H&E staining of formalin-fixed, paraffin-embedded sections of a brain and a spinal cord harvested from EBV, EBV+CsA and PBS control groups. Dotted circles point to well-demarcated cellular aggregates. 40×, 10× and 5× images show scale bar of 20μm, 100μm, and 200μm, respectively.

To evaluate the impact of peripheral EBV infection on the CNS, we examined the brain and spinal cord for histopathological changes. Interestingly, we observed widespread presence of distinct cellular aggregates consisting of inflammatory cells and microglia nodules in the brain and spinal cord of (2/8) EBV and (7/9) EBV+CsA groups (**Figure 2**). These aggregates were not observed in any of the PBS controls. However, 2/3 CsA controls developed similar CNS aggregates. These observations suggest that peripheral EBV infection can promote neuroinflammation in some hosts, and is likely influenced by the host’s genetics and immune system. Immunosuppression can also lead to neuroinflammation, possibly as a result of reactivation of latent infection(s) other than EBV. We also noted that the cellular aggregates in the spinal cord were less in number and smaller in size than cerebral aggregates (**Figure 2**). Moreover, the aggregates in the brain were widespread and present throughout the hemisphere, including the meninges and the cerebellum. Generally, these cellular aggregates were associated with CNS vasculature (**Supplementary Figure 2A**). The aggregates were observed more frequently in the cerebrum than in the cerebellum. The aggregates also formed in both hemispheres (**Supplementary Figure 2B**).

**Figure 3:**
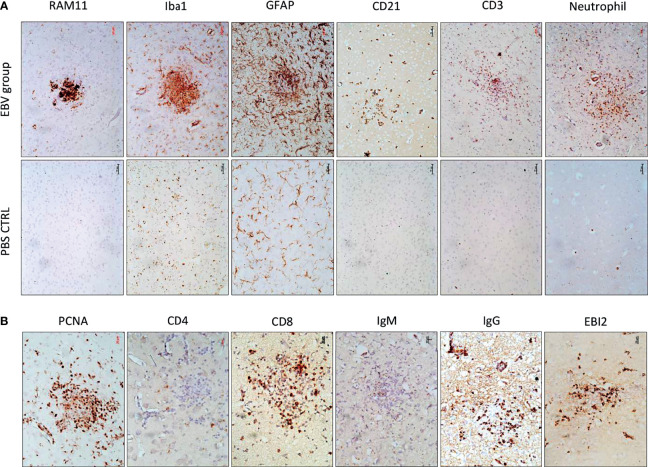
The cellular makeup of cerebral aggregates in EBV infected rabbits. **(A)** Brain sections from EBV infected rabbits with cerebral aggregates and PBS controls were stained with rabbit-specific maker for macrophages (RAM11), microglia (Iba1), astrocytes (GFAP), B cells (CD21), T cells (CD3), and neutrophils. Scale bar=50μm. **(B)** Additional phenotypic characterization of lymphoid infiltrates by staining for the proliferation marker PCNA, T and B cell markers CD8, IgG, IgM, and Epstein-Barr virus-induced gene 2 (EBI2). Scale bar=20μm.

**Figure 4:**
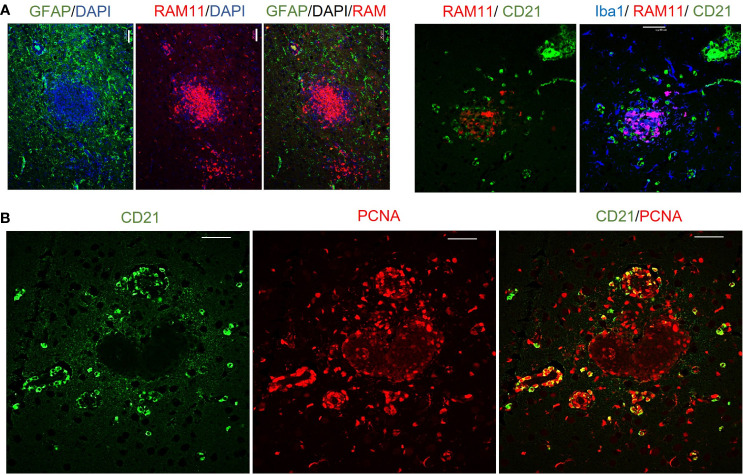
Immunofluorescence double staining to determine the cellular makeup of cerebral aggregates in EBV infected rabbits. **(A)** Representative immunofluorescence staining of inflamed brain section with GFAP (green), RAM11 (red), and DAPI (blue). Scale bar=20μm. Location of B cells (green) in relation to macrophages (red) and microglia (blue) were identified by staining for CD21, RAM11 and Iba1, respectively. Scale bar=40μm. **(B)** Double positive CD21 (green) and PCNA (red) depict proliferating B lymphocytes in cerebral aggregates. Scale bar=40μm.

**Figure 5:**
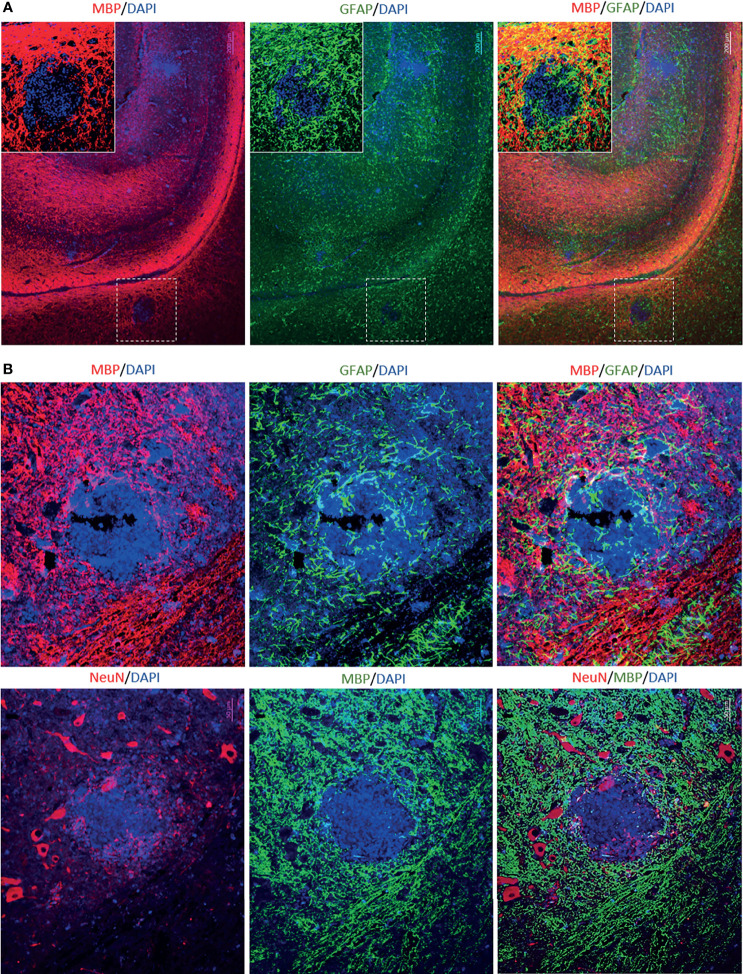
Immunofluorescence double staining to assess demyelination in aggregates-positive case from EBV group. **(A)** Immunostaining for MPB and GFAP in the brain. Scale bar=200μm **(B)** Immunostaining for MBP, GFAP and NeuN in the corresponding spinal cord. The aggregates were completely devoid of myelinated nerve fibers. However, demyelination appeared to be restricted to the aggregates and it was not widespread. Scale bar=50μm.

To determine the cellular makeup of the CNS aggregates, we immunostained sections of the brain and spinal cord for various cellular markers. CNS aggregates consisted of infiltrating macrophages (RAM11^+^), microglia (Iba1^+^), reactive astrocytes (GFAP^+^), and infiltrating B (CD21^+^) and T lymphocytes (CD3^+^), and neutrophils (**Figure 3A** and **Supplementary Figure 3**). Additionally, proliferating cells (PCNA^+^) and infiltrating lymphocytes, including CD8^+^ (but not CD4^+^), IgG^+^ (but not IgM^+^) and EBI2^+^ cells were dispersed within the aggregates (**Figure 3B**). Notably, the majority of these infiltrating immune cells were also diffusely scattered in the CNS parenchyma and formed several small clusters of loosely connected cells that lacked macrophage aggregation. While blood-derived macrophages/microglia appeared to make up the center of most, if not all, CNS aggregates, astrocytes and scattered proliferating B cells were mainly associated with the outer part of the aggregates (**Figures 4A, B**). Furthermore, to examine whether the presence of these aggregates is associated with demyelination, we stained for myelin basic protein (MBP). We observed disruption of myelin within the aggregates, but this did not extend beyond the aggregates (**Figure 5**). Collectively, these observations suggest that peripheral EBV infection can lead to immune cells trafficking into the CNS, and the formation of cellular aggregates. The aggregates consist of proliferating B cells, T-cells, and astrocytes, with blood derived macrophages occupying the center. Notably, the aggregates appear to be completely devoid of myelinated nerve fibers.

#### 3.1.3 EBV Infected Cells Infiltrate the Brain of Both Immunocompetent and Immunosuppressed Animals

**Figure 6:**
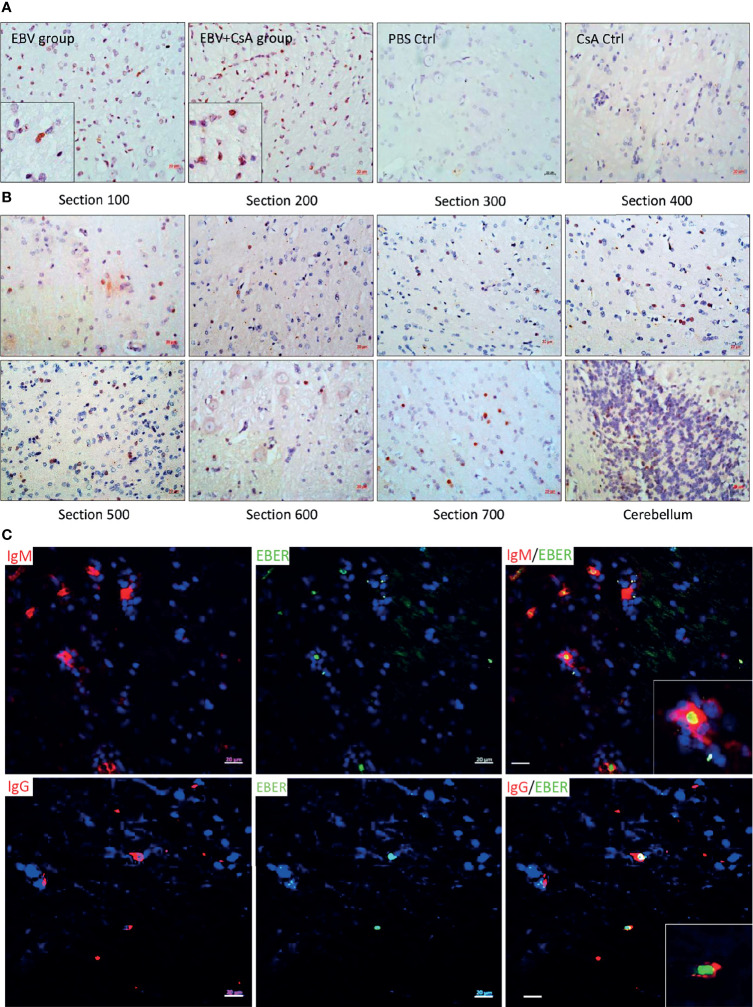
EBV infected cells infiltrated the brain. **(A)** Representative images of EBER-ISH on brain sections from EBV and EBV+CsA groups and their corresponding PBS/CsA controls. Scale bar=20µm. **(B)** Immunohistochemistry for EBNA1 in a series of sections from a heavily infected cerebral hemisphere and cerebellum. Scale bar=20µm. **(C)** Double staining for IgG/IgM (red) and EBERs (green) to determine the phenotype of infected cells in the brain. Scale bar=20µm.

Since EBV infection of the brain has previously been shown in MS cases (9–11, 18, 23–27), we wanted to know whether primary peripheral infection can lead to infection of the brain. We stained brain sections, from EBV and EBV+CsA groups and PBS/CsA controls, for EBERs. EBV was detected in the brain of 6/8 animals in the EBV group, and 9/9 in the EBV+CsA group, independent of the presence of cellular aggregates. The 2/8 animals negative for EBV in the brain, contained no cellular aggregates. EBV infected cells were not seen in any of the PBS/CsA controls (**Figure 6A**). We also examined the distribution of transcriptionally active virus in the brain by staining a series of sections with anti-EBNA1. EBNA1^+^ cells were seen dispersed throughout the brain (**Figure 6B**). Remarkably, massive infiltration of EBNA1^+^ cells took place in the granular layer of the cerebellum (**Figure 6B**), suggesting that the cerebellum may be a vulnerable niche to EBV infection in the CNS (28). However, further work is required to evaluate the importance of the cerebellum in EBV infection.

In humans, EBV is primarily carried by circulating IgD^-^ CD27^+^ isotype-switched memory B cells (29, 30). To determine which cells are infected with EBV in the rabbit brain, we performed double staining for EBV (EBERs) and B-cells (IgM and IgG), in heavily infected brain sections. Both IgM^+^ and IgG^+^ cells were found to be EBV positive (**Figure 6C**). These findings indicate that primary peripheral EBV infection can be a sufficient event for EBV infected B cells to infiltrate the CNS.

### 3.2 Investigating the Dynamics of EBV Infection Over Time

#### 3.2.1 EBV Load Peaks at Day 14 Post Infection in Peripheral and CNS Compartments

To evaluate infection dynamics and delineate changes in the incidence of CNS aggregates over time, we IV inoculated a new batch of rabbits with EBV and examined blood, spleen, brain, and spinal cord at five time points; 3, 7, 14, 21 and 28 days post infection (dpi).

**Figure 7:**
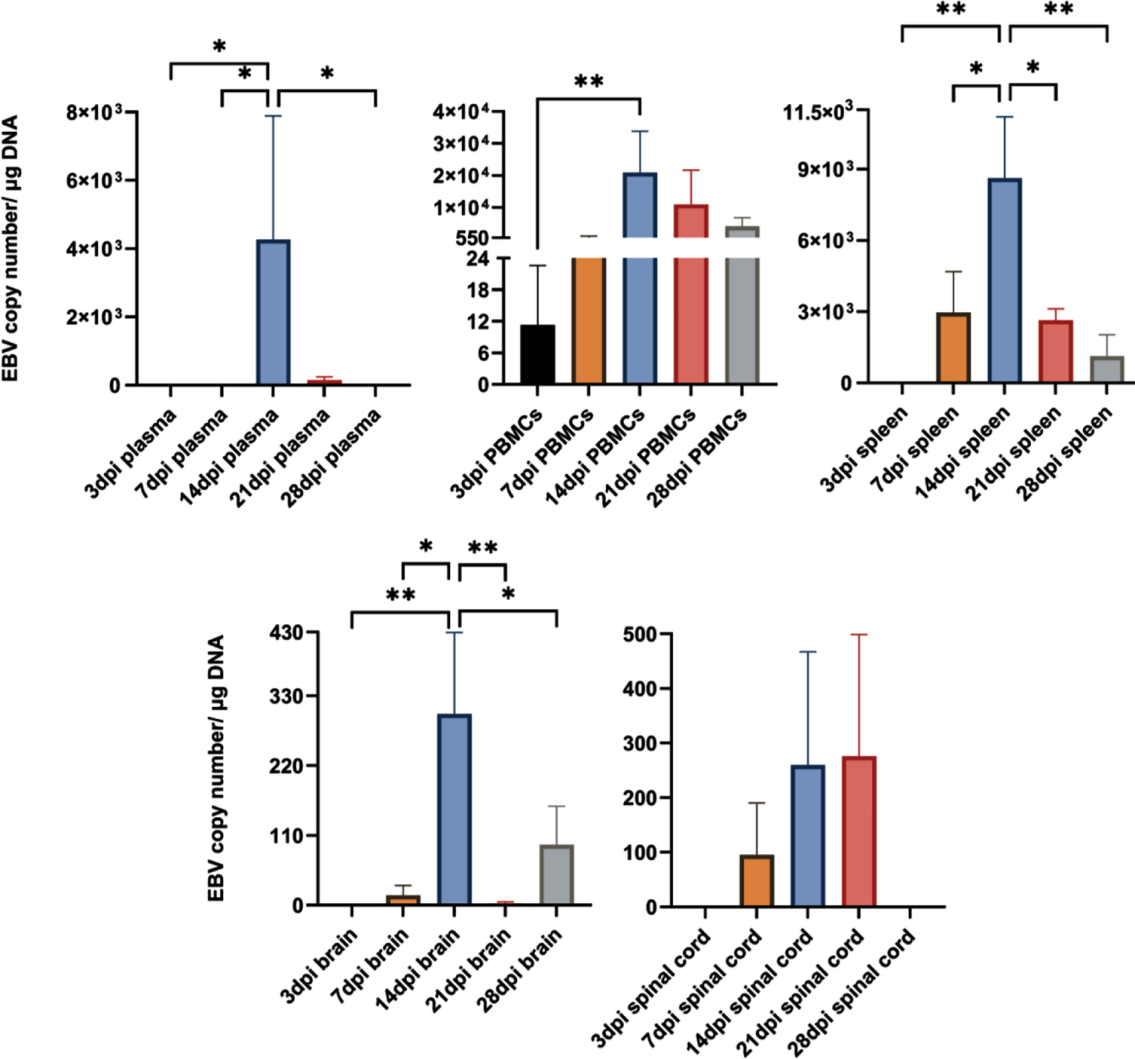
EBV load peaks in the periphery and CNS at 14dpi. EBV copy number in plasma, PBMCs, spleen, brain and spinal cord determined by qPCR at 3, 7, 14, 21 and 28dpi (*n*=3 rabbits/time-point), and displayed as mean ± SEM. One-Way ANOVA and non-parametric Kruskal-Wallis test were used to compare between groups. *p ≤ 0.05, **p ≤ 0.01. Bars without asterisks are not significantly different.

In the peripheral compartment, EBV was detected in the PBMCs at all time points (**Figure 7**). In the spleen, however, virus reached detectable levels by day 7 and remained detectable throughout the next 3 time points. Interestingly, EBV in the plasma (indicative of viremia) could not be detected until 14 and 21dpi. In the CNS, the virus was detected in the brain at 7, 14, 21 and 28dpi, and in the spinal cord at 7, 14 and 21dpi. Notably, all 3 animals (100%) sacrificed at day 14 exhibited detectable high virus load in the plasma, PBMCs, spleen and brain. Thus, day 14 was the optimal time point for virus detection in the periphery and CNS. Additionally, EBV DNA load increased significantly at day 14 in plasma, PBMCs, spleen and brain (**Figure 7**). Furthermore, EBV DNA load in the brain correlated significantly with both splenic and PBMCs viral load (**Supplementary Table 3**). However, EBV load in the spinal cord correlated only moderately with EBV levels in the plasma (viremia) (**Supplementary Table 3**). Thus, increased viral load, in the spleen and PBMCs, may be a determinant for virus infection of the brain. This also highlights the importance of cell-associated virus, rather than free virus, in EBV trafficking to the brain.

#### 3.2.2 The Occurrence of CNS Aggregates Peaks at Days 14 and 21 of Infection

**Figure 8:**
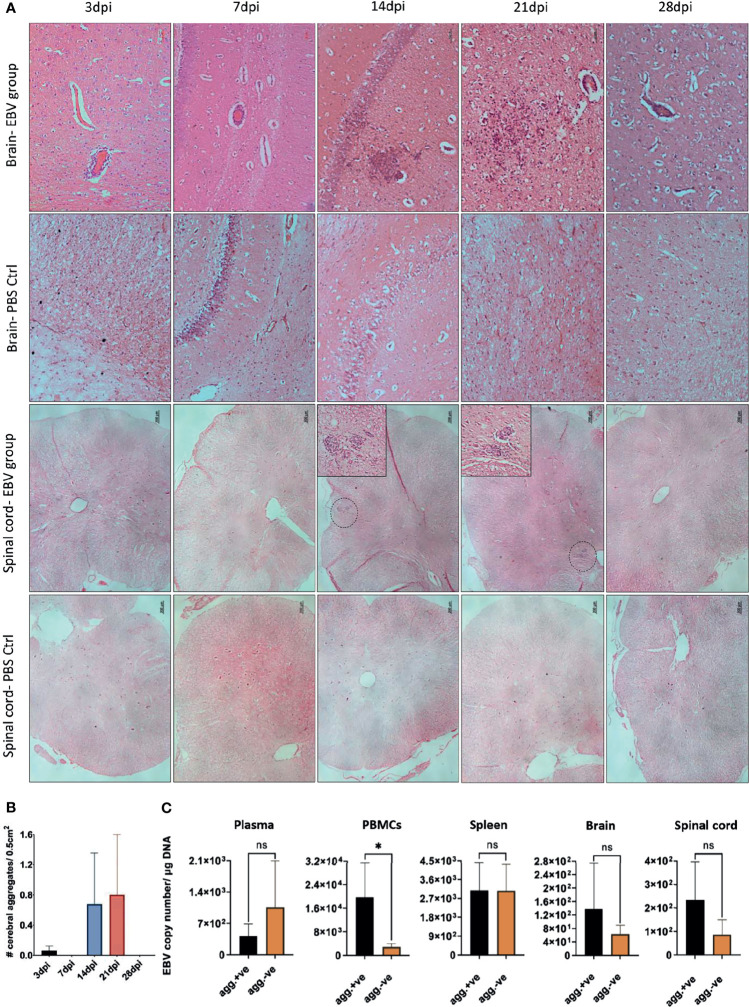
CNS aggregate formation peaks at 14 and 21dpi. **(A)** Representative images of H&E staining of coronal brain sections (scale bar=50µm) and cross sections of spinal cord (scale bar=200µm) from EBV group and PBS control group (PBS ctrl) sacrificed at 3, 7, 14, 21 and 28dpi. **(B)** Quantification of cell aggregates in the brain. Aggregates were counted in one every 50 5µm-sections over a span of ~1000 brain sections from EBV infected animals sacrificed at 3, 7, 14, 21 and 28dpi (*n*=3 rabbits/time-point). Number of cell aggregates per 0.5cm^2^ are displayed as mean ± SEM. Comparisons were made using the non-parametric Kruskal-Wallis test. **(C)** Comparison of EBV load in plasma, PBMCs, spleen, brain and spinal cord of animals with CNS aggregates and those without using two-tailed unpaired t test and Mann-Whitney test. ns: *p >* 0.05, *p ≤ 0.05.

**Figure 9:**
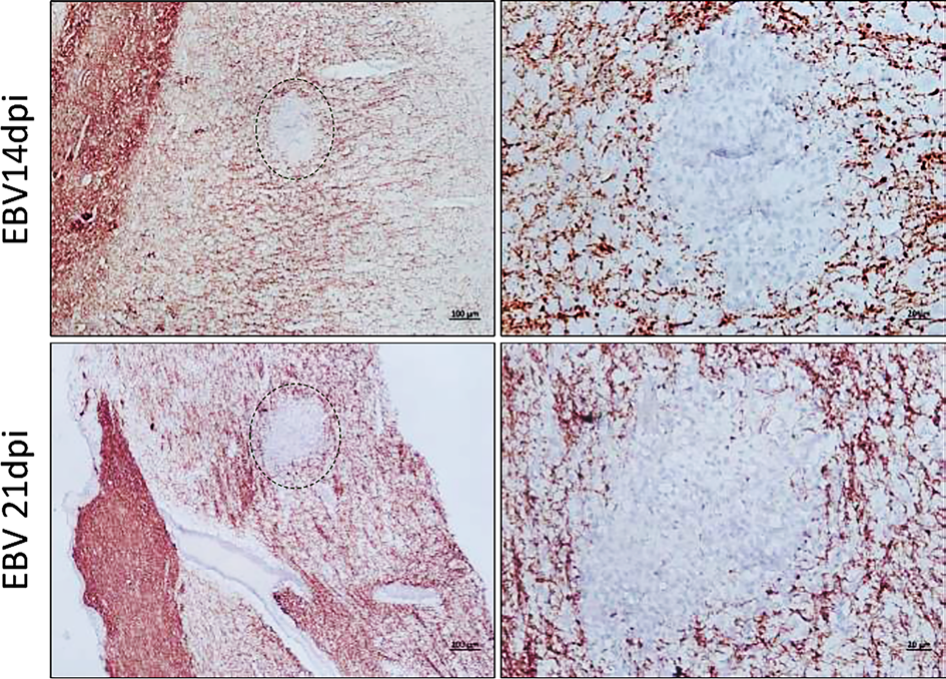
Inflammatory aggregates in the EBV infected brain are free of myelinated nerve fibers. Brain sections were examined for demyelination in and around the aggregates by immunostaining for MBP. Demyelination was evident within aggregates developed at 14dpi and 21dpi. Scale bar at lower magnification=100µm. Scale bar at higher magnification=20µm.

We next examined coronal sections of the brain and cross sections of the spinal cord for the presence of inflammatory aggregates at 3, 7, 14, 21, and 28dpi. Although mild inflammation was frequently observed in the meninges and around blood vessels in the brain of infected animals, distinct cellular aggregates were only seen in the brain and spinal cord of animals sacrificed at days 14 and 21 (**Figure 8A**). Again, the aggregates in the spinal cord were smaller in size compared to the aggregates observed in the corresponding brain. Additionally, massive infiltration of the CNS by immune cells, including neutrophils, macrophages (**Supplementary Figure 4**), and B and T lymphocytes (**Supplementary Figure 5**) was observed at days 14 and 21 in sections with aggregates, but not in sections with limited mild inflammation. In agreement with part 1 of the study, the formation of aggregates was associated with disruption of myelin within aggregates, in both the brain (**Figure 9**) and spinal cord (**Supplementary Figure 6**).

To determine if there was a difference in the number of cerebral aggregates at different time points, we cut 1000, 5µm sections from all animals at each of the five time points. Aggregates were counted in sections at intervals of 50. Aggregates were observed to have great heterogeneity in morphology. Thus, for the purpose of counting, we defined an aggregate in the brain parenchyma as a clear continuous cluster of cells that is at least 60µm in diameter. The term aggregate also included any 2 cellular clusters that were connected by a thread of infiltrates. Thus, meningeal infiltrates, small vessels engorged with lymphocytes, clusters or perivascular cuffs that are less than 60µm in diameter were not counted. On average the number of aggregates reached the peak at 14 and 21dpi (**Figure 8B**). We next compared peripheral and CNS viral load in animals that developed CNS aggregates and those without aggregates. We found that animals with aggregates had significantly elevated levels of EBV DNA in PBMCs compared to animals without aggregates (**Figure 8C**). This reflects a link between the level of cell-associated virus, but not cell-free virus, in the peripheral blood and the occurrence of CNS cellular aggregates. Thus, viremia may not be a determinant for the development of these structures in the CNS.

#### 3.2.3 Peripheral EBV Infection Results in Altered Expression of Cytokines and Latent Viral Transcripts in the Brain

**Figure 10:**
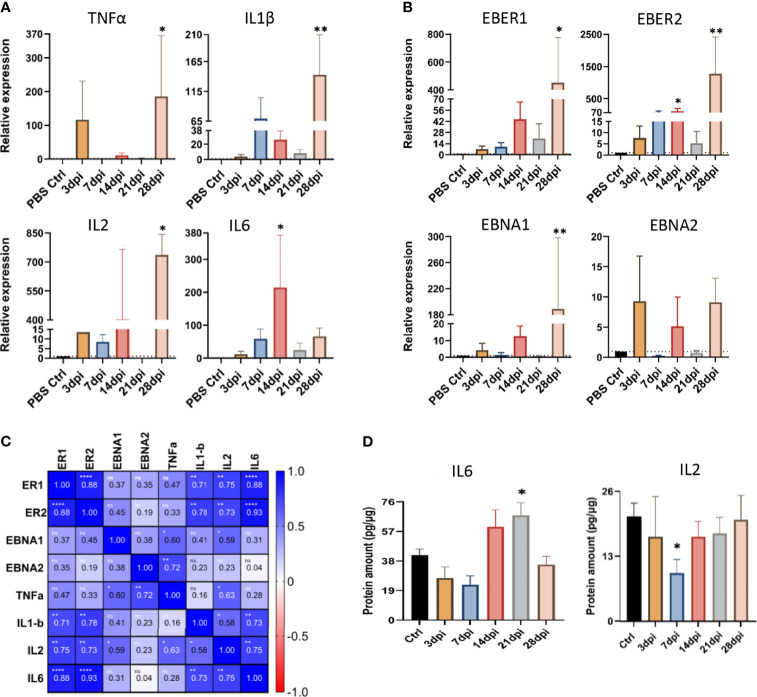
Peripheral EBV infection results in altered expression of cytokines and viral transcripts in the brain. The expression of **(A)** cytokines (TNFα, IL1β, IL2, and IL-6), and **(B)** EBV latent transcripts (EBER1, EBER2, EBNA1 and EBNA2), in the brain tissue harvested at 3, 7, 14, 21, and 28dpi, examined using qPCR. Data displayed as mean ± SEM. Comparisons were made using one-way ANOVA with Fisher’s LSD test or the nonparametric Kruskal-Wallis test. **(C)** Heat map of Spearman correlation of inflammatory and viral transcripts in the brain. Color mapping for positive and negative correlation are indicated in the legend on the right. ns: p >0.05, *p ≤ 0.05, **p ≤ 0.01, ****p ≤ 0.0001. **(D)** The levels of IL2 and IL6 proteins were measured by ELISA in brain tissues harvested at 3, 7, 14, 21 and 28dpi. Data displayed as mean ± SEM. Comparisons were made using one-way ANOVA with Fisher’s LSD test or Welch and Brown-Forsythe test. *p ≤ 0.05, **p ≤ 0.01 compared to PBS controls. Bars without asterisks are not significantly different from the PBS controls.

To understand the impact of peripheral EBV infection on the expression of proinflammatory cytokines in the brain, we performed qPCR for tumor necrosis factor α (TNFα), interleukin-1β (IL1β), interleukin-2 (IL2) and interleukin-6 (IL6). Analysis of relative expression over the 5 time points of infection revealed significant upregulation of TNFα, IL1β and IL2 at 28dpi, compared to the brain of non-infected PBS controls (**Figure 10A**). IL6, on the other hand, was significantly upregulated as early as 14dpi (**Figure 10A**). Furthermore, we determined whether the upregulation of these cytokines in the brain was coupled with altered expression of EBV latent transcripts. Similar to TNFα, IL1β and IL2, the expression of EBER1, EBER2 and EBNA1 was significantly elevated at day 28 (**Figure 10B**). EBER2, however, was significantly upregulated at day 14. This shows that the expression of proinflammatory cytokines correlate with that of viral transcripts in the brain.

Indeed, heat map of Spearman correlation of viral transcripts and cytokine expression in the brain, indicated a positive correlation between EBER1/2 and the cytokines IL6, IL2 and IL1β, (**Figure 10C**). Together, these results imply that increased mRNA expression of latent EBV transcripts is accompanied by increased expression of inflammatory cytokines in the brain.

We also examined the impact of EBV infection on the protein levels of IL2 and IL6 in the brain. Both 14 and 21dpi showed dramatic rise in the levels of IL6 protein, yet it only reached statistical significance at day 21, coinciding with the time points of aggregate occurrence (**Figure 10D**). Interestingly, there was a significant drop in the regulatory IL2 level at day 7 (**Figure 10D**), the time point that preceded aggregate formation. These observations suggest that the increased production of proinflammatory IL6 may be associated with the inflammation seen in the brain.

## 4 Discussion

Some studies have found no indication of EBV infection in the MS brain (31–33). Whereas others have demonstrated the presence of the virus in the brain, implicating a role for the virus in the pathogenesis of this disease (9, 11, 18). However, the dynamics of virus trafficking to the brain, and its subsequent impact on disease development and/or progression, are poorly understood. Addressing these questions has been challenging due to the limited availability of an animal model that recapitulates the typically silent-mild infection seen in most humans. We and others have recently shown that rabbits are susceptible to EBV, and the infection mimics that observed in humans (16, 34–36). Previous studies have demonstrated the efficacy of intravenous (IV) inoculation in producing persistent infection in rabbits (35–37). This route of infection was shown to elicit antiviral humoral response and detectable virus and viral proteins in peripheral blood, spleen, and liver. Additionally, EBV levels in the periphery varied between different rabbits, and fluctuated overtime in a given animal (37). Similar to humans, the rabbit immune system does not completely eradicate EBV infection, as these animals remain latently infected with the virus (36). Attempts to infect rabbits *via* intranasal route were also successful, however, this was found to lead to lower expression of EBV proteins and milder infection compared to IV inoculation. Using the oral route, on the other hand, appeared less effective in establishing infection (35). Based on these observations, we used IV route in this study to ensure establishment of persistent EBV infection in rabbits.

Using the rabbit model, we explored the neuropathogenic potential of primary peripheral EBV infection. The findings uncovered several novel aspects of the dynamics of EBV infection in the periphery and CNS. 1) Intravenous inoculation of the virus resulted in widespread infection in all three peripheral compartments examined: spleen, PBMCs, and plasma. 2) Peripheral infection resulted in the virus traversing the brain. 3) Infection in the brain correlated with cell-associated virus, rather than circulating free virus in the plasma. 4) Peripheral EBV infection induced the formation of inflammatory cellular aggregates in the CNS, and these aggregates were composed of blood-derived macrophages surrounded by reactive astrocytes and infiltrating B and T lymphocytes.

Primary EBV infection during late adolescence can cause symptomatic infectious mononucleosis (IM). Both symptomatic and asymptomatic primary infection cause high viral load in the periphery. However, disrupted immunological profile is rather unique to IM (38–40). This emphasizes that EBV associated diseases emerge as a result of changes in the immune components triggered by the infection. Indeed, abnormalities in anti-EBV immune response exerted by CD8^+^ T cells is believed to contribute to MS disease (41–43). In this study, we investigated primary EBV infection in healthy rabbits, and rabbits immunosuppressed with CsA. High viral load was detected in the peripheral compartments of all animals, particularly the immunosuppressed (EBV+CsA) group. This group also exhibited more than 10-fold higher levels of free virus in the plasma. However, the level of free virus did not correlate with brain infection. By contrast, there was a positive correlation between infected cells in the PBMCs/spleen, and brain infection. These findings support the idea that CNS infection is due to migrating infected lymphocytes, most probably B cells. van Langelaar and coauthors have recently shown that there is a positive correlation between IgM^-^ IgD^-^ B cells expressing the chemokine CXCR3 and EBV load in the blood of MS patients who underwent bone marrow transplantation (44). This may mechanistically implicate this chemokine in the migration of virus infected cells to the CNS. Furthermore, EBV infected B cells with phosphoprotein 1/osteopontin gene upregulation have been found to have the potential of infiltrating the CNS (45). The gene upregulation in these cells is associated with epigenetic changes including histone modification. The migration of EBV infected cells from the periphery to the brain has also been reported recently in humanized mice (46). This was achieved by inoculating humanized mice with EBV and treating them with pembrolizumab, a monoclonal antibody, used clinically to block the immune checkpoint programmed death 1 (PD-1) receptor. Subsequently, virus propagation to the CNS led to the formation of EBER-rich lymphomas in the brain. These mice had low frequency of circulating T cells, many of which were exhausted (i.e. TIM3^+^ and LAG3^+^ T cells) (46).

Different animals have been used to study EBV infection in the brain. Some studies investigated intracerebral inoculation of MHV68 into mice (15, 47, 48). Animals exhibited signs of severe disease, which was more fatal in juvenile mice than in older ones (48). The direct introduction of the virus into the CNS was shown to result in mononuclear cell infiltrates and viral infection of the meninges, ependymal cells, oligodendrocytes, hippocampal pyramidal neurons, and the Bergmann glia cells in the cerebellum. The infection was also associated with damage to the white matter (47). Japanese macaques were also found to naturally develop an acute MS-like disease as a result of CNS infection with a newly identified γ-herpesvirus. The CNS contained several inflammatory demyelinating lesions (49). The CNS infiltrating CD4^+^ T cells and CD8^+^ T cells were later shown to elicit immune response against the myelin antigens MBP, myelin oligodendrocyte glycoprotein and proteolipid protein (50). Rhesus monkeys were also reported to develop inflammation in the brain (infiltration of T lymphocytes and macrophages in the parenchyma and meninges) when they were administered autologous B lymphoblastoid cell lines infected with a γ-herpesvirus pulsed *ex vivo* with MOG peptides (51).

In our study, immune cell aggregation developed in rabbit brains without overt signs of neurological deficits. Similarly, it has been reported that intranasal infection of 129/SvEv mice with rabies CVS-F3 does not result in neurological manifestations, despite the occurrence of neuroinflammation, BBB breakdown and the increased expression of the proinflammatory cytokines such as IL6 and TNFα (52). Importantly, cell aggregates formed only in some animals. Why only a fraction of infected animals developed CNS aggregates remain to be explored. However, our results suggest that EBV load in PBMCs may partly be linked to the formation of these structures. Additionally, host factors such as genetic background and the fitness of immune system to control viral infection are also likely to be important. HLA alleles are believed to interact with EBV to shape disease susceptibility in people with MS (53, 54), while peripheral EBV load is found to correlate positively with the MS risk allele HLA-DRB1*15 and negatively with the protective allele HLA-A*02 (55). Moreover, EBV latent protein EBNA2 is thought to interact with risk loci related to MS and other autoimmune diseases (56). Addressing the effect of HLA-DRB1*15, EBV infected humanized mice reconstituted with HLA-DR15+ immune system components were shown to exhibit poor control over the virus despite the increased activation and proliferation of T lymphocytes (57). Remarkably, some T lymphocytes from these animals were found to cross-react with the MBP (57).

Cell aggregates in the CNS of rabbits contained a heterogeneous cell population made up of brain resident cells, infiltrating macrophages, neutrophils and B and T lymphocytes. In general, aggregates were seen at dissimilar stages of evolution in a given section, and thus differed in composition. Most of the aggregates had infiltrating macrophages as the prominent core surrounded by reactive astrocytes and dispersed lymphocytes. However, few aggregates lacked macrophage infiltration, but contained either a cluster of reactive glia or loosely connected lymphocytes. Brain-infiltrating T lymphocytes were mainly CD8^+^ cells. The scarcity of CD4^+^ cells within aggregates cannot be simply due to the effect of CsA, because the number of CD4^+^ cells was also limited in aggregates formed in the EBV group. Only few CD4^+^ cells were scattered in the parenchyma. The data suggested that the contribution of CD4^+^ cells to both CNS infiltration and aggregate formation was minimal.

Immune aggregates reminiscent of organized lymphoid structures were previously recognized in the meninges of MS brain, and have gained attention as a potential pathogenic feature of the disease (58, 59). In addition to MS, EBV infection has been associated with the formation and/or persistence of these immune aggregates (also known as ectopic lymphoid-like structures) in the inflamed tissue in certain organ-specific autoimmune diseases (9, 60–62). The ectopic lymphoid-like structures observed in meningeal inflammation in MS contained distinct clusters of CD20^+^ B cells and CD138^+^ plasma cells, intermingled with CD35^+^ follicular dendritic cells and CD3^+^ T cells (63). These structures expressed markers that determine the fate of B cells including CXCL13, CD27, and BAFF (25, 64). In rabbits, B lymphocytes contributed to the meningeal inflammation and aggregate formation in brain parenchyma. However, the cell organization and phenotypes observed in inflammation in rabbits did not mimic the typical organization of ectopic lymphoid-like structures reported in MS. B lymphocytes in the rabbit aggregates expressed proliferation marker PCNA, IgM, IgG and EBI2. Notably, EBI2 has been reported to be upregulated in activated T and B lymphocytes, and affects the movement of these cells (58–61). EBI2 expression by astrocytes was shown to promote the migration of macrophages (62). Moreover, the cellular aggregates observed in the rabbit CNS were entirely devoid of myelinated nerve fibers, suggesting that some form of demyelination was occurring within these aggregates. However, the underlying mechanisms for this demyelination remain to be further investigated.

We also observed that viral load peaked at day 14 post infection, both in the peripheral and CNS compartments. EBV load in peripheral blood, but not in CNS, correlated with aggregate formation. Small sample size could be one possible explanation for not seeing a significant difference in the CNS viral load between animals that developed aggregates and those that did not. Alternatively, it may be possible that the formation of aggregates is influenced by the expression of EBV transcripts and not the viral load. We noticed increased expression of IL6 mRNA and protein in aggregate positive brain at day 14 of infection. Moreover, IL6 expression strongly correlated with EBV-encoded EBERs. In agreement with our results, the viral load of Theiler’s Murine Encephalomyelitis Virus (TMEV) in the CNS of mice has been shown not to correlate with the development of experimental autoimmune encephalitis (EAE) (65). Instead, disease outcome correlated well with immune response to viral components. Thus, virus trafficking into the CNS is not sufficient for the neuropathological changes to occur.

Another important finding from our rabbit model is the positive strong correlation between increased expression of viral latent transcripts, particularly the viral RNAs, EBERs, and the cytokines IL1β, IL6, and IL2 in the brain. By contrast, the lytic transcript BZLF1 could not be detected in the brain with or without aggregates at any of the time points, ruling out the role of the lytic cycle in inflammation. In some animals in EBV+CsA, however, BZLF1 was detected in few cells in the brain. On similar grounds, induction of EAE in mice infected with MHV-68 was shown to result in aggravated disease (66). The onset of disease course of EAE coincided with the virus establishing latency in mice, and not during the acute pre-latent infection. Mice that were infected with latency deficient MHV68 had significantly milder disease than those latently infected with the wild-type virus. The latent infection in mice was found to cause increased T lymphocyte infiltration into the CNS, and suppress the anti-inflammatory phenotype of T cells; regulatory T cells, both in the periphery and CNS (66).

We also observed a positive correlation between the expression of *EBNA1* and *EBNA2* and *TNFα* expression in the brain. A recent study reported that immunizing mice with EBNA1 amino acid region 411-426 led to neurological deficits reminiscent of EAE, and the development of MRI-confirmed cortical lesions (67). This region of EBNA1 was also found to trigger high antibody response in individuals with relapsing-remitting and secondary progressive MS, and these antibodies cross-reacted with MBP amino acid region 205-224 (67). Furthermore, EBV latent proteins were found to be upregulated in MS lesions (27). Virus reactivation in the MS brain was also associated with marked neuroinflammation and demyelination leading to fatal immune reconstitution inflammatory syndrome (68, 69). Our study and these reports support the hypothesis that transcriptionally active EBV in the brain promotes immunological alterations.

Additionally, we demonstrated elevated mRNA levels of IL1β and TNFα at the later stage of infection (28dpi). These Th1 cytokines (IL1β, and TNFα, IFNγ) were implicated in impaired BBB (52, 70–73). It has been suggested that virus infection of the CNS incites the generation of inflammatory cytokines, which in return compromises the integrity of BBB, for example by altering the expression of BBB tight junction proteins (74–77). Thus, BBB breakdown could be a consequence of viral infection of the CNS (74, 76, 78). One could argue that increased mRNA levels of IL1β, and TNFα at 28dpi may be followed by increased BBB permeability and recurrent influx of immune cells into the CNS. Whether EBV infection disrupts BBB integrity warrants further investigation.

Another critical issue arising from this study is the need to determine antigen specificity of lymphoid infiltrates in the CNS. The functional characterization of virus-specific immune response could further explain the inflammatory response and identify the extent of the damage brought about by either virus infected cells or immune response directed against transcriptionally active virus (79). It has been shown that EBV-specific CD8^+^ T cells make up ~0.5-2.5% of total brain-infiltrating CD8^+^ T cells in MS (23). This frequency was found to be significantly higher than CD8^+^ T cells reactive against MBP, CMV, or influenza virus. Further characterization of EBV-specific CD8^+^ T cells showed the expression of degranulation marker CD107a, perforin, and granzyme B, indicating their cytotoxic nature (23).

## 5 Conclusions

In conclusion, our results support a neuropathogenic potential of EBV. The neuroinflammation and immunopathological aspects of EBV gleaned from the rabbit model will help us explore, otherwise poorly understood, viral-host interactions that can be essential for the pathogenesis of EBV-associated neuropathologies including MS. The flexibility of this model offers avenues to examine the CNS-periphery axis during viral infection, and to identify potential cofactors for EBV-associated neuropathology. Further studies are needed to determine the cellular behavior and events that are crucial in the formation of neuroinflammatory aggregates, the resulting tissue damage, and the resolution of inflammation. Studying this cascade of events can provide us with an opportunity to critically evaluate potential and specific therapeutic targets that are essential to either halt the progression of EBV-associated neuropathologies or promote resolution of neuroinflammation.

## Data Availability Statement

The original contributions presented in the study are included in the article/**Supplementary Material**. Further inquiries can be directed to the corresponding author.

## Ethics Statement

The animal study was reviewed and approved by Animal Research Ethics Committee of UAE University (Approval numbers: A-15-15; ERA-2018-5718).

## Author Contributions

Study conception and design was performed by GK. AH and NR performed animal experiments, sample collection, and data analysis and interpretation. SS provided technical guidance on histopathology examination, immunohistochemistry and immunofluorescence staining. AH and GK drafted the manuscript. All authors contributed to the article and approved the submitted version.

## Funding

This work was funded by UAEU Zayed Centre-Based grants 31R135 and 31R259.

## Conflict of Interest

The authors declare that the research was conducted in the absence of any commercial or financial relationships that could be construed as a potential conflict of interest.

## Publisher’s Note

All claims expressed in this article are solely those of the authors and do not necessarily represent those of their affiliated organizations, or those of the publisher, the editors and the reviewers. Any product that may be evaluated in this article, or claim that may be made by its manufacturer, is not guaranteed or endorsed by the publisher.
