# Supplementary material for: Primary Peripheral Epstein-Barr Virus Infection Can Lead to CNS Infection and Neuroinflammation in a Rabbit Model: Implications for Multiple Sclerosis Pathogenesis
Source: Front Immunol. 2021 Nov 25;12:764937. doi: 10.3389/fimmu.2021.764937 (PMC8656284; doi:10.3389/fimmu.2021.764937)
Supplement: Supplementary file 1 [file DataSheet_1.pdf]

Supplementary Material

1 Supplementary Figures

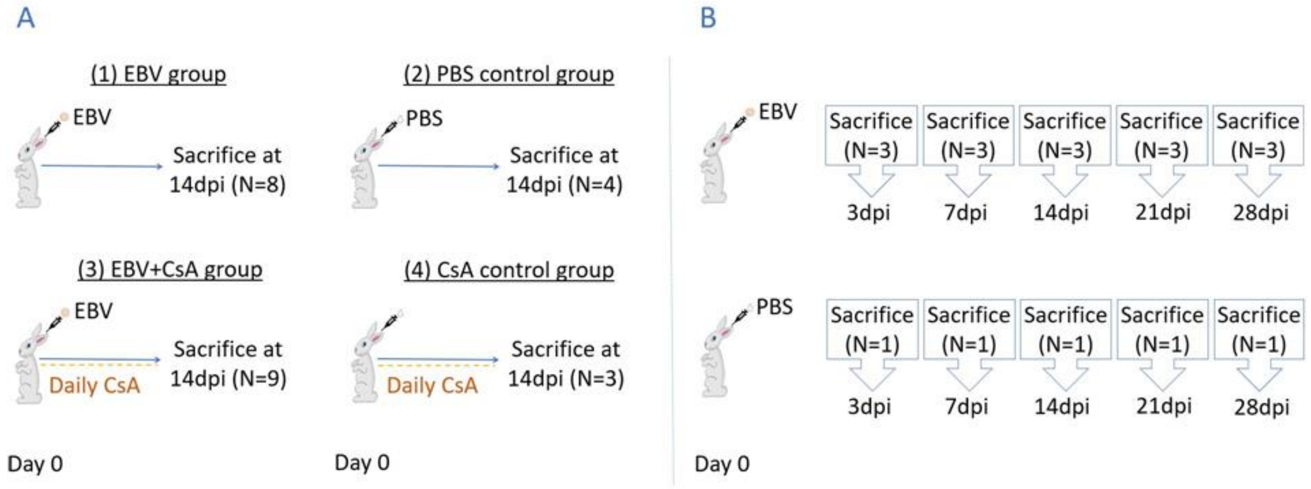

Supplementary Figure 1. The experimental design of the study. (**A**) Part 1 of the study (investigating viral spread from the periphery to the CNS) was carried out on four groups: EBV (*n*=8), PBS controls (*n*=4), EBV+CsA (*n*=9), and CsA controls (*n*=3). All groups were sacrificed at 14dpi. (**B**) Part 2 of the study (investigating dynamics of EBV infection over time) was carried out on two groups: EBV and PBS controls. Three animals from EBV group and one PBS control were sacrificed at each of the following five time points: 3, 7, 14, 21 and 28dpi

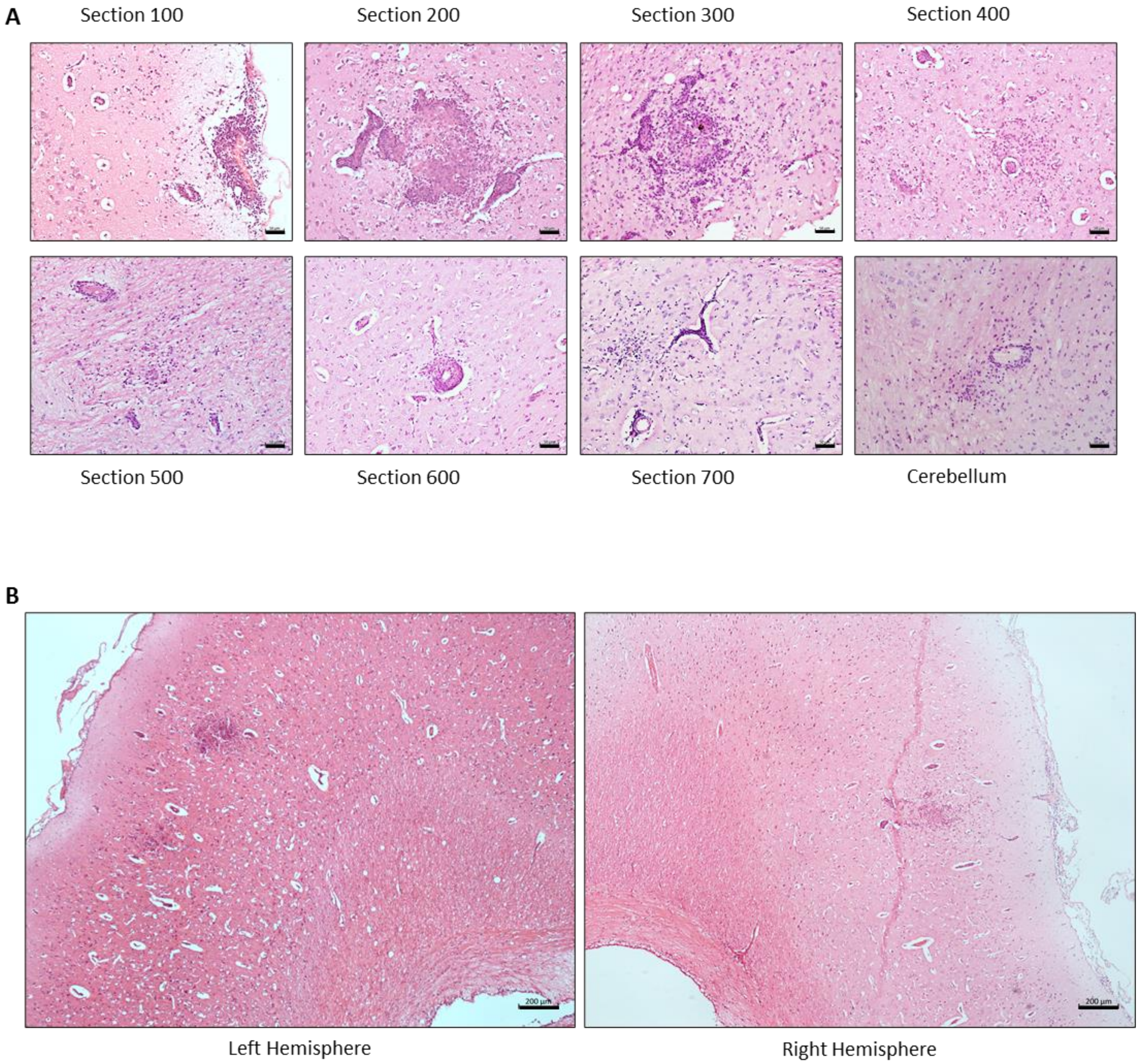

Supplementary Figure 2. Widespread cerebral aggregates. (**A**) Representative H&E staining of series of sections from a brain hemisphere with cell aggregates. Scale bar= 50µm. (**B**) Representative H&E staining of left and right hemispheres from the brain of an EBV infected rabbit that developed cerebral aggregates. Scale bar= 200µm

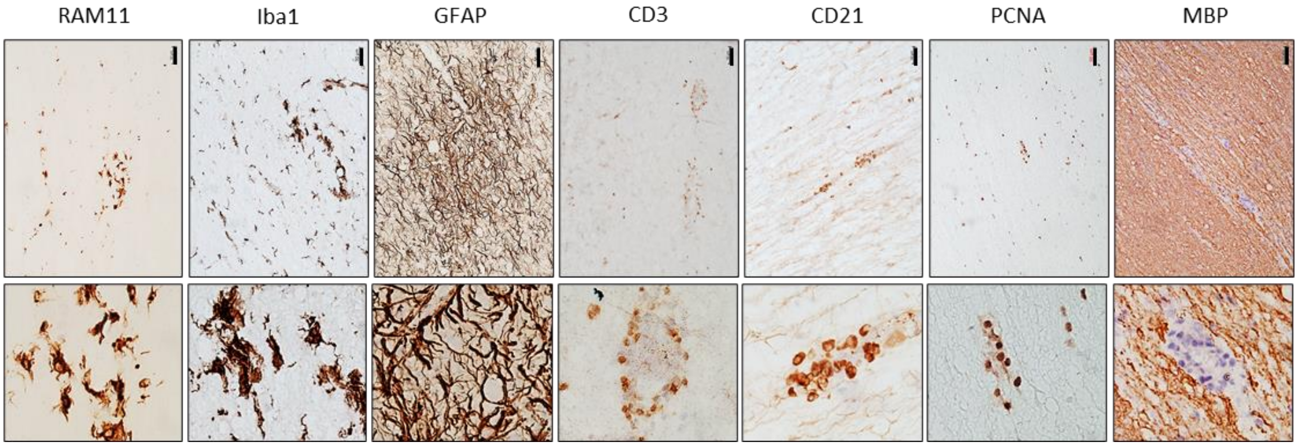

Supplementary Figure 3. The cellular makeup of spinal cord inflammatory infiltrates in EBV infected rabbit. FFPE spinal cord sections were stained with rabbit-specific macrophage marker RAM11, microglia marker Iba1, astrocyte marker GFAP, T cell marker CD3, B cell marker CD21, proliferation marker PCNA and MBP. Scale bar= 50μm

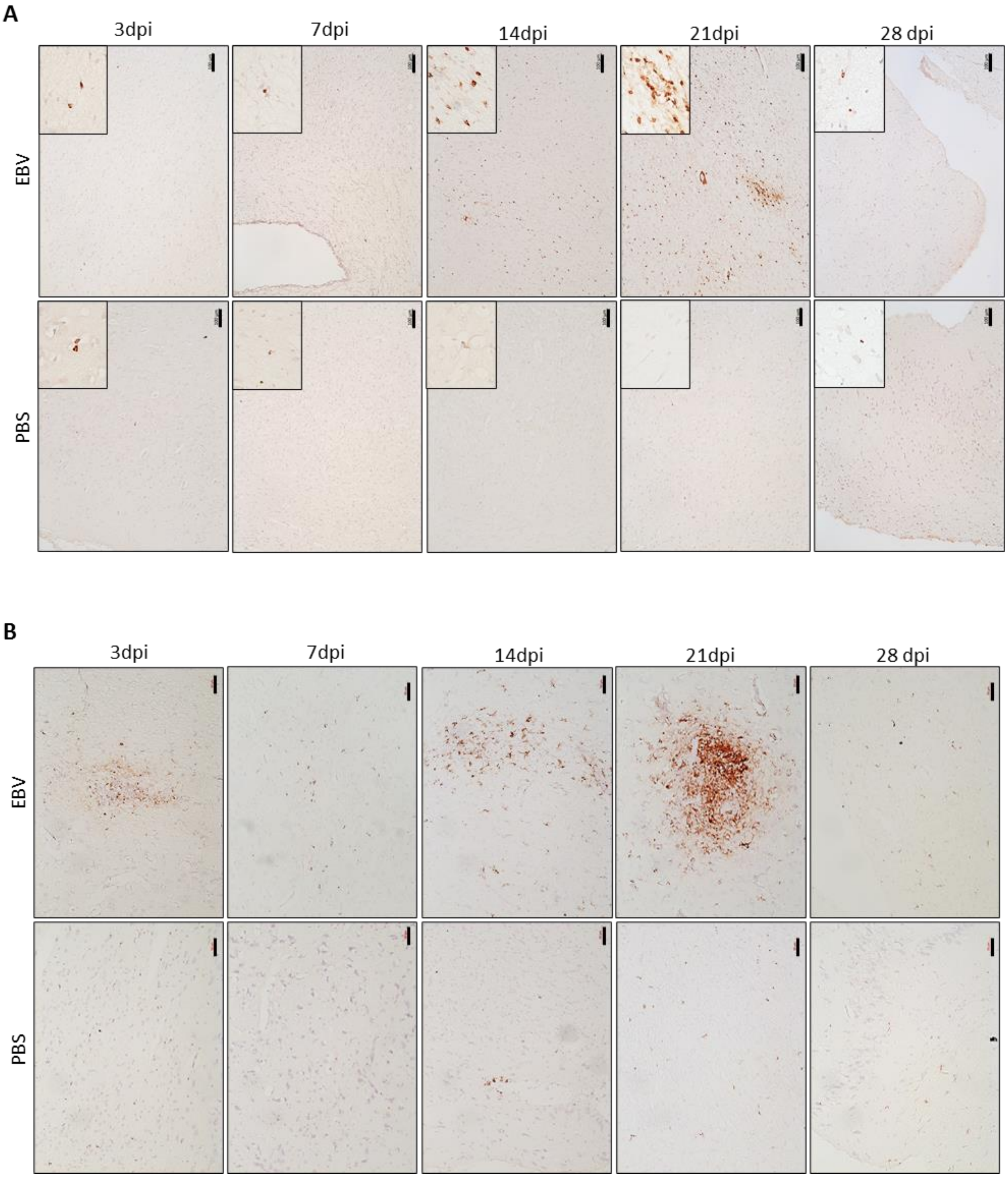

Supplementary Figure 4. Infiltration of neutrophils and macrophages became prominent in aggregate positive brain sections at 14 and 21dpi. (**A**) Immunohistochemistry staining for rabbit-specific neutrophil marker in EBV group and PBS controls. Scale bar=100µm. (**B**) Immunohistochemistry staining for rabbit-specific vascular macrophages; RAM11, in EBV group and PBS controls. Scale bar=50µm

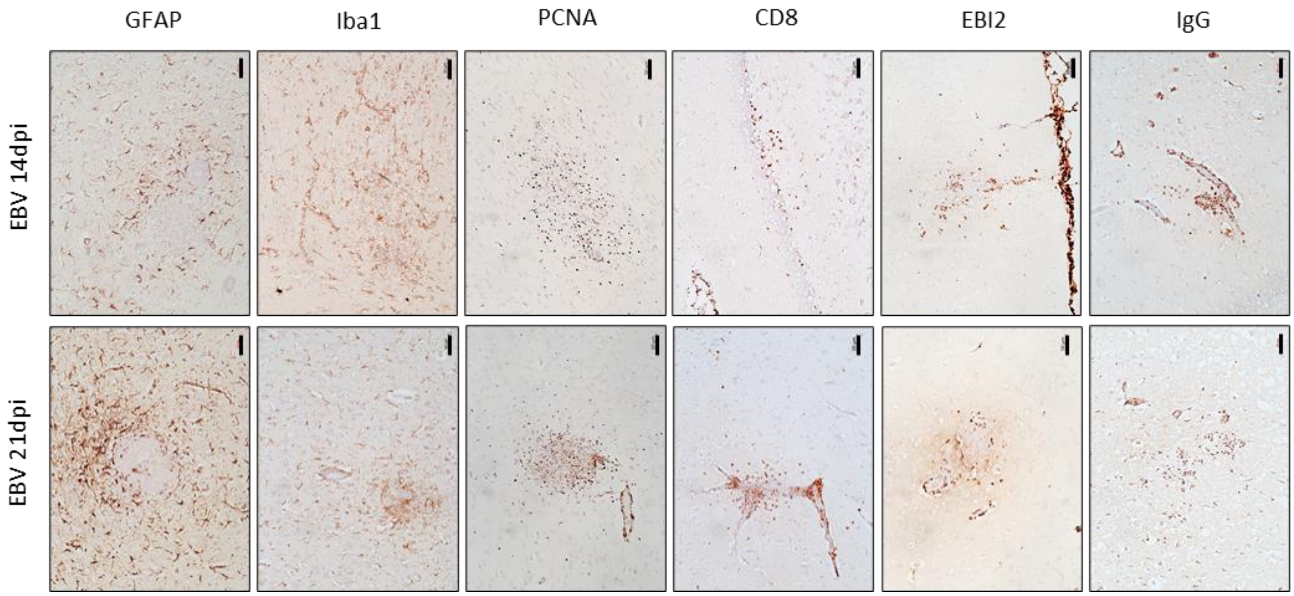

Supplementary Figure 5. Reactive glia and infiltrating B and T lymphocytes in the brain, with signs of demyelination. Aggregate positive brain sections from animals sacrificed at 14 and 21dpi were stained for GFAP (astrocyte marker), Iba1 (microglia marker), PCNA (proliferation marker), CD8 (CD8+ T cell marker), EBI2 (chemotactic receptor for lymphocytes), and IgG (marker for isotype-switched B lymphocytes). Scale bar=50µm

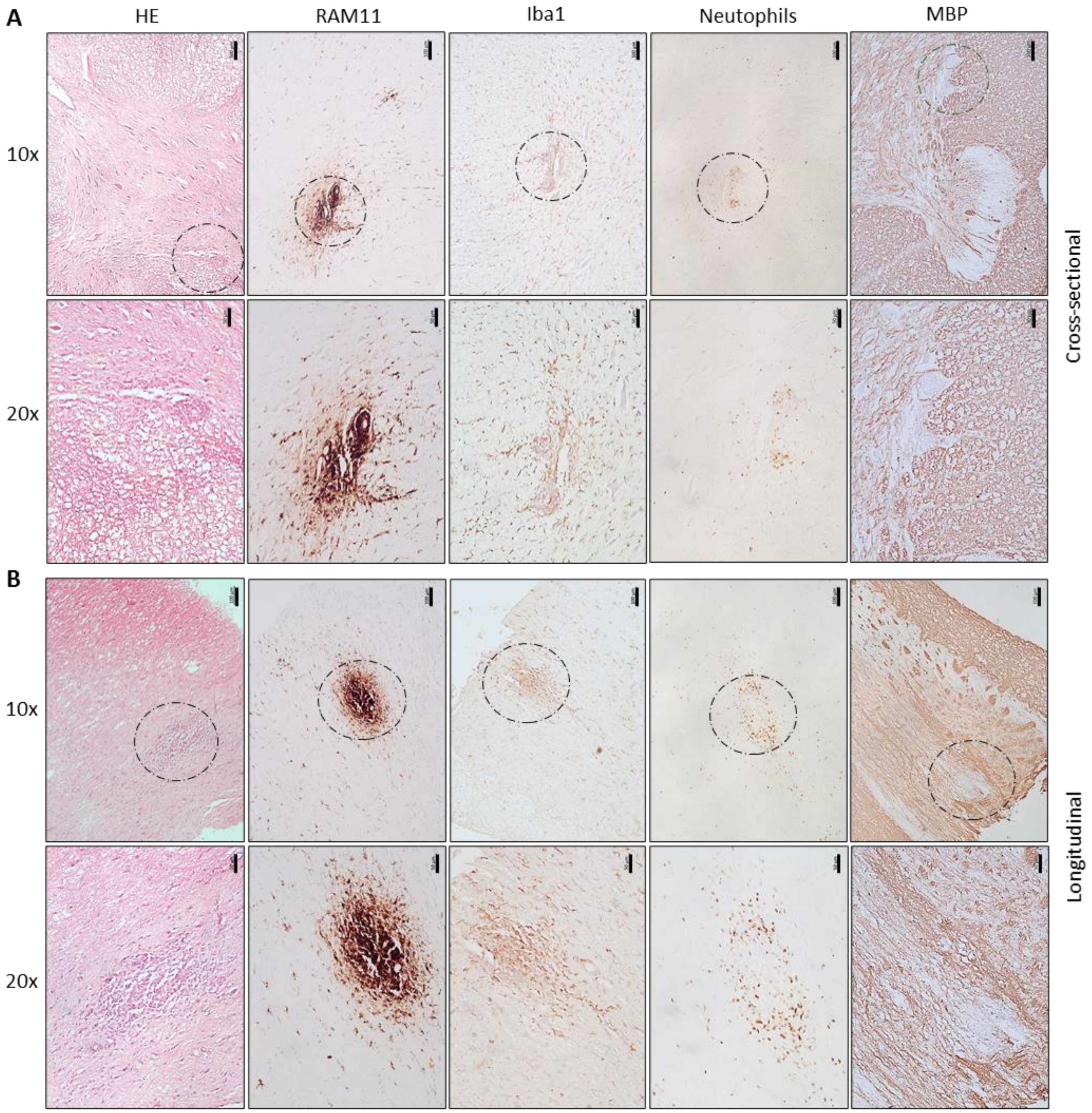

Supplementary Figure 6. Infiltration of the spinal cord by macrophages and neutrophils. Cross-sectional **(A)** and longitudinal **(B)** sections of a spinal cord positive for aggregates stained with H&E, and for RAM11 (rabbit-specific vascular macrophages), Iba1 (microglia marker), rabbit-specific neutrophil marker and myelin basic protein (MBP). Scale bar at lower magnification=100µm. Scale bar at higher magnification=50µm

2 Supplementary Tables

Unconjugated Antibodies	Host species	Dilution- IHC	Dilution- IF	Source	Clone
Anti-CD3	Rat	1:250	-	ab11089/ Abcam	[CD3-12]
Anti-CD4	Mouse	1:200	-	WS0778U-100/ KingFisher Biotech	[RTHA]
Anti-CD8	Mouse	1:400	-	WS0796U-100/ KingFisher Biotech	[ISC27A]
Anti-CD21	Rabbit	1:250	1:250	ab75985/ Abcam	[EP3093]
Anti-DIG	Mouse	1:5000	-	Sigma	[D1-22]
Anti-EBI2	Rabbit	1:300	-	ab150625/ Abcam	polyclonal
Anti-EBNA1	Mouse	1:20	-	MA1-7271/ Thermo	[D810H]
Anti-GFAP	mouse	1:1000	-	G3893/ Sigma	[G-A-5]
Anti-GFAP	Chicken	-	1:500	ab4674/ Abcam	polyclonal
Anti-Iba1	Goat	1:2000	1:100	ab5076/ Abcam	polyclonal
Anti-Rabbit IgM mu chain	Goat	1:2000	1:1000	ab97191/ Abcam	
Anti-Rabbit IgG Fc	Goat	1:3000	1:500	ab190492/ Abcam	[RMG02]
Anti-rabbit macrophages	Mouse	1:300	1:50	M0633/ Dako	[RAM11]
Anti-MBP	Rat	1:100	-	ab7349/ Abcam	[12]
Neutrophil marker	Mouse	1:200	-	sc-59376/ Santa Cruz	[RPN3/57]
Anti-PCNA	Mouse	1:10000	1:100	ab29/ Abcam	[PC10]
Fluorochrome-conjugated antibodies	**Host species**	**Dilution**		**Source**	**Clone**
Anti-DIG-FITC	Mouse	1:250		F3523/ Sigma	[D1-22]
Anti-Chicken IgY-FITC	Goat	1:200		(ab6873/ Abcam	polyclonal
Anti-Goat IgG- Rhodamine Red™-X (RRX)	Donkey	1:100		Code: 705-295-147/ Jackson ImmunoResearch	polyclonal
Anti-Goat IgG-Alexa Fluor® 647	Donkey	1:100		Code: 705-607-003/ Jackson ImmunoResearch	polyclonal
Anti-mouse IgG-Alexa Fluor® 555	Goat	1:100		4409S/ Cell Signaling	
Anti-rabbit IgG-Alexa Fluor® 488	Goat	1:200		4412S/ Cell Signaling	

Supplementary Table 1. List of antibodies used in the study

	EBV+CsA plasma vs. EBV+CsA PBMCs	EBV+CsA plasma vs. EBV+CsA spleen
Spearman r	0.2204	0.147
P (two-tailed)	0.4677	0.6323
	EBV plasma vs. EBV PBMCs	EBV plasma vs. EBV spleen
Pearson r	0.2646	0.7884
P (two-tailed)	0.5265	0.0201

Supplementary Table 2. The relationship between load of cell-free EBV (in plasma) and viral load in PBMCs and spleen. The Correlation of viremia levels with EBV load in PBMCs and spleen using Spearman correlation for EBV+CsA group, and Pearson correlation for EBV group

	EBV load in the brain vs. EBV load in plasma	EBV load in the brain vs. EBV load in PBMCs	EBV load in the brain vs. EBV load in spleen
Spearman r	0.4128	0.686	0.7212
	0.1278	0.0065	0.0037
P (two-tailed)			
	EBV load in the spinal cord vs. EBV load in plasma	EBV load in the spinal cord vs. EBV load in PBMCs	EBV load in the spinal cord vs. EBV load in spleen
Spearman r	0.5582	0.2499	0.3797
P (two-tailed)	0.0202	0.3655	0.1623

Supplementary Table 3. Spearman correlation of EBV load in the CNS and viral load in the peripheral compartments (plasma, PBMCs and spleen). Correlation coefficient (*r*) and p value are indicated
